# Function evaluation of the autoanalyser internal quality control of the Kehua Polaris c2000 automatic biochemical analyser

**DOI:** 10.5937/jomb0-47151

**Published:** 2024-11-16

**Authors:** Yiyi Wang, Lei Shi, Jue Zhang, Chen Chen

**Affiliations:** 1 Shanghai Shuguang Hospital Affiliated with Shanghai University of TCM, Department of Laboratory Medicine, Shanghai, China

**Keywords:** Polaris c2000 automatic biochemical analyser, clinical biochemical detection, internal quality control, turnaround time, automatski biohemijski analizator Polaris c2000, klinička biohemijska detekcija, unutrašnja kontrola kvaliteta, vreme obrade

## Abstract

**Background:**

The present study aims to (1) evaluate the autoanalyser quality control (QC) module of the Kehua Polaris c2000 automatic modularised biochemical analyser and (2) verify the impact of the analyser's automatic implementation of internal QC (IQC) testing at a set time point on the results of IQC and turnaround time (TAT).

**Methods:**

For 5 consecutive days, three different methods were used to conduct IQC. Method 1: Internal QC was carried out at 8:00 every day. Method 2: The QC products were placed in the instrument calibration QC plate in the afternoon, and the instrument was set to measure the products at 7:00 the next day automatically. Method 3: The QC products were placed in the instrument calibration QC plate, and the instrument was set up for automatic measurement at 7:30 every day. All three methods were compared and evaluated. The effect of IQC on the TAT was monitored using method 2.

**Results:**

There were no statistical differences in the IQC results between methods 2 and 1. However, there were statistical differences in some items between methods 3 and 1; thus, the IQC results of method 2 can be adopted. Implementing method 2 for IQC can help achieve a significant TAT-saving of 35.23 min during the automatic retest process after the IQC indicates an out-of-control situation. This time reduction is highly valuable for improving efficiency and streamlining the testing workflow.

**Conclusions:**

Using the autoanalyser QC module of the Kehua Polaris c2000 automatic modular biochemical analysis system to perform IQC has no impact on the IQC test results and can save TAT, as well as automatically correct most out-of-control occurrences.

## Abstract (sr)

**Uvod:**

Ova studija ima za cilj da (1) izvrši evaluaciju modula za kontrolu kvaliteta (QC) automatskog modularnog biohemijskog analizatora Kehua Polaris c2000 i da (2) verifikuje uticaj automatskog sprovođenja unutrašnje kontrole kvaliteta (IQC) analizatora u određeno vreme na rezultate IQC i vreme obrade (TAT).

**Metode:**

Tokom 5 uzastopnih dana su korišćene tri različite metode za sprovođenje unutrašnje kontrole kvaliteta (IQC). Metod 1: Unutrašnja kontrola kvaliteta se sprovodila svakog dana u 8:00. Metod 2: QC proizvodi su postavljani na kalibracionu QC ploču instrumenta u popodnevnim satima, a instrument je podešen da automatski meri proizvode sledećeg dana u 7:00. Metod 3: QC proizvodi su postavljani na kalibracionu QC ploču instrumenta, a instrument je podešen za automatsko merenje svakog dana u 7:30. Sve tri metode su upoređene i evaluirane. Efekat IQC na vreme obrade (TAT) je praćen korišćenjem metode 2.

**Rezultati:**

Nije bilo statistički značajnih razlika u rezultatima unutrašnje kontrole kvaliteta (IQC) između metoda 2 i 1. Međutim, postojale su statističke razlike u nekim stavkama između metoda 3 i 1, te stoga rezultati IQC iz metode 2 mogu da budu prihvaćeni. Primena metode 2 za IQC može da pomogne u smislu smanjenja vremena obrade (TAT) za 35,23 minuta tokom automatskog ponovnog testiranja nakon što IQC ukaže na situaciju koja nije pod kontrolom. Ovo smanjenje vremena je veoma značajno za poboljšanje efikasnosti i optimizaciju toka testiranja.

**Zaključak:**

Korišćenje QC modula autoanalizatora Kehua Polaris C2000 automatskog modularnog sistema za biohemijsku analizu za izvođenje sprovođenje kontrole kvaliteta (IQC) nema uticaja na rezultate IQC testova i može da skrati vreme obrade (TAT), kao i da automatski ispravi većinu nepravilnosti.

## Introduction

With the development of medical science, medical laboratories play a crucial role in diagnosing, treating, preventing diseases and assessing health status. Laboratory quality control (QC) plays a central role in ensuring the accuracy and reliability of laboratory test results. Quality management of QC covers the whole experiment process, including preparation before, operation during, and evaluation after analysis. The purpose is to control the experimental conditions, operators, equipment, and test methods and to establish and follow the established control criteria to ensure the test results’ quality [1].

As key equipment in a clinical laboratory, automatic biochemistry analysers can perform various biochemical tests on biological samples such as blood, serum and plasma, which is indispensable in modern health care. Such analysers significantly improve the accuracy and precision of the test by precisely controlling the amounts of samples and reagents, reaction time and temperature, signal acquisition, and result analysis while substantially improving the efficiency of the test [2]. A large automatic biochemical analyser is the main equipment for laboratory biochemical tests, and its internal QC (IQC) results are the main tools to determine whether the test results are accurate.

In this study, the autoanalyser QC module of the Kehua Polaris c2000 automatic modular biochemical analysis system was applied. This study aimed to evaluate the performance of the internal quality control (IQC) module of the Corva Polaris c2000 and investigate the impact of its automated implementation of IQC testing on IQC results and sample test turnaround time (TAT). The study aimed to determine whether automated IQC testing could reduce the impact of human operations and improve the accuracy and efficiency of the test while verifying its specific impact on IQC results and TAT, providing laboratories with a more efficient method to use the system.

## Materials and methods

### Instruments and reagents

The Kehua Polaris c2000 automatic modular biochemical analysis system and a reagent were purchased from Shanghai Kehua Biological Engineering Co., Ltd. The detection speed of the system is 2,000 tests per hour. The QC products (L1/L2) were purchased from Roche Diagnostic Products (Shanghai) Co., Ltd. Details of the reagents, calibrations and QC products used in this study are shown in Table 1, and the QC rules are in in-control.

**Table 1:** Specifications of the reagents, calibrations, and quality control products.

Name	Manufacturer	Method	Lot number	Date of<br>manufacture	Period of validity
Routine chemical quality	Roche		32419608	20191002	20210130
control PCCC1	Diagnostic				
Routine chemical quality	Roche		16038603	20191108	20210130
control PCCC2	Diagnostic				
Kehua calibrator	Kehua		2019031K	20190316	20210315
Amylase (AMY)	Kehua biology	EPS Substrate Method	20200118	20200109	20210119
Total protein (TP)	Kehua biology	Biuret Method	20190612	20190611	20201230
Albumin (ALB)	Kehua biology	Bromocresol Green Method	20200218	20200113	20210630
Triglyceride (TG)	Kehua biology	GPO-PAP Method	2018123C	20181213	20201230
Cholesterol (CHOL)	Kehua biology	CHOD-PAP Method	20190213	20190210	20200913
Alanine Transaminase	Kehua biology	UV-LDH Method	20200318	20200311	20210801
(ALT)					
Aspartate	Kehua biology	UV-MDH Method	2020021K	20200216	20210425
aminotransferase (AST)					
Alkaline phosphatase	Kehua biology	AMP Buffer Method	20191013	20191015	20201025
(ALP)					
γ-Glutamyltransferase	Kehua biology	L-γ-glutamyl-3-carboxy-<br>4-nitroanilide Method	20191012	20191021	20201101
(γ-GT)					
Creatine kinase (CK)	Kehua biology	NAC Method	2019011D	20190115	20210101
Lactate dehydrogenase	Kehua biology	Lactate Method	20200418	20200420	20210425
(LDH)					
Urea nitrogen (BUN)	Kehua biology	UV-GLDH Method	20190718	20190720	20211015
Uric acid (UA)	Kehua biology	Uricase-PAP Method	2019031R	20190315	20200920
Creatinine (CREA)	Kehua biology	Sarcosine Oxidase-PAP<br>Method	2019071R	20190715	20201025
Calcium (Ca)	Kehua biology	Arsenazo Colorimetric<br>Method	20191111	20191121	20201125
Phosphorus (P)	Kehua biology	UV Method	2019081K	20190815	20201210

### Methods

### Internal quality control analysis

The QC products (L1/L2) were dissolved in accordance with the instructions, using three different methods to perform IQC. 3 replicates per sample per day. The coefficient of variation of all item results was <1/4 of the allowable total error [3]. All samples were run in randomised order each day by each method to reduce bias.

Method 1: After opening the bottle and dissolving the products, the QC products were kept in a refrigerator at 2~8 °C. At 8:00 each day, 400 mL of the QC products were drawn, placed into a microcup, and repeatedly tested thrice for 5 consecutive days.

Method 2: After opening the bottle and melting the products, the QC products were kept in a refrigerator at 2~8 °C. Next, 400 μL of the QC product was placed into a micro-cup at 16:00 every day, and the cup was placed on the IQC plate. The instrument was set to repeatedly (three times) and automatically detect at 7:00 the following day for 5 consecutive days.

Method 3: After opening the bottle and dissolving the products, 3 mL of the QC products were placed in the biochemical tube and placed on the IQC plate. The instrument was set to repeatedly (three times) and automatically detect at 7:00 the following day for 5 consecutive days.

In Methods 2 and 3, QC samples were selected to be placed at 16:00 p.m. and then tested at 7:00 a.m. the following morning to mimic the scenario where the laboratory prepares samples at the end of the workday and automatically tests using night-time. This reduces operation during working hours, increases efficiency, and allows IQC testing to be completed before staff arrives.

The IQC judgment rules are 13S, 22S, R4S, 41S and 8’X. The allowable total error reference was WS/T 403-2012 Analytical quality specifications for routine analytes in clinical biochemistry [3]. QC samples are frozen prior to analysis to avoid potential biases due to factors such as sample evaporation.

### Turnaround time monitoring

The IQC detection was performed using method 2, as described previously. The TAT of the first samples of the day for two consecutive months (45 working days) (TAT in the laboratory; the time between the laboratory receiving the samples and issuing the report) was monitored and compared with that of the IQC results obtained using method 1.

### The correction rate of automatic retest for runaway after IQC

During the two months (45 working days) of IQC using method 2, the total runaway number of 16testing items and the number of corrections through automatic retesting and QC were counted. Furthermore, the correction rate was calculated.

### Statistical analysis

Statistical analysis was performed using Microsoft Office Excel 2013 and the SPSS Statistics 18.0 software to complete the calculation of the mean, standard deviation and coefficient of variation. If data were normally distributed, the ± SD was used with a t-test for comparison. The rank sum test was used if the data had a skewed distribution. If *p* < 0.05, the difference was statistically significant.

## Results

### Internal quality control results using three methods for 5 consecutive days

According to the IQC judgment rules (13S, 22S, R4S, 41S and 8), the IQC data of all 16 items tested using method 1 were not out of control, and the coefficient of variation of all item results was <1/4 of the allowable total error. The IQC data of the items tested by methods 2 and 3 were not out of control, and the coefficient of variation was <1/4 of the allowable total error [4]. The detection results of the three methods are detailed in Table 2. The data had either normal or approximately normal distribution and were analysed using a t-test.

**Table 2:** 5-day test data of Roche conventional chemical QC products. Note: # indicates a statistically significant difference compared with Method 1 (P <0.05).

		Quality			
Order<br>number		Control product	Method 1	Method 2	Method 3
		level			
1	Albumin (g/L)	PCCC1	38±0.27	38±0.58	37±0.55^#^
		PCCC2	55±0.45	55±0.60	54±0.71^#^
2	Cholesterol (mmol/L)	PCCC1<br>PCCC2	2.79±0.03<br>6.24±0.08	2.80±0.04<br>6.24±0.07	2.70±0.06^#^<br>6.10±0.09^#^
3	Triglyceride (mmol/L)	PCCC1	1.56±0.02	1.58±0.02	1.54±0.03^#^
		PCCC2	2.93±0.05	2.96±0.03	2.90±0.06
4	Urea nitrogen (mmol/L)	PCCC1	8.06±0.15	8.12±0.21	7.97±0.29
		PCCC2	22.12±0.36	22.37±0.48	22.16±0.46
5	Uric acid (μmol/L)	PCCC1	335±5.15	339±4.57	333±5.99
		PCCC2	747±8.66	751±9.11	749±13.10^#^
6	Creatinine (μmol/L)	PCCC1<br>PCCC2	108±1.40<br>425±5.12	109±1.67<br>426±4.85	113±2.08^#^<br>434±6.29^#^
7	Glutamic oxaloacetic<br>transaminase (U/L)	PCCC1	61±0.62	61±0.74	60±0.60
		PCCC2	170±1.39	170±1.01	170±0.93
8	Alanine<br>aminotransferase (U/L)	PCCC1	57±0.23	57±0.69	57±0.71
		PCCC2	147±0.71	147±1.02	147±0.96
9	Creatine kinase (U/L)	PCCC1	179±1.90	179±1.76	179±2.36
		PCCC2	354±4.53	354±3.17	353±4.57
10	Isocitrate<br>dehydrogenase (U/L)	PCCC1	180±4.08	181±3.33	184±6.31^#^
		PCCC2	326±7.12	326±7.11	330±5.69^#^
11	Amylase(U/L)	PCCC1	107±1.00	108±0.87	109±1.53^#^
		PCCC2	246±2.17	246±2.80	247±3.11
12	Alkaline phosphatase<br>(U/L)	PCCC1	116±2.23	117±1.47	116±2.58
		PCCC2	258±5.17	260±4.04	257±3.93
13	γ-Glutamyl<br>transpeptidase (U/L)	PCCC1	52±0.56	53±0.39	51±0.68^#^
		PCCC2	274±2.23	274±1.67	271±2.67^#^
14	Total protein (g/L)	PCCC1	57±0.53	57±0.54	57±1.34
		PCCC2	87±0.83	87±1.20	86±0.83^#^
15	Calcium (mmol/L)	PCCC1	2.59±0.03	2.60±0.04	2.51±0.05^#^
		PCCC2	3.74±0.04	3.76±0.04	3.68±0.04^#^
16	Phosphorus (mmol/L)	PCCC1	1.52±0.02	1.52±0.02	1.4±0.02^#^
		PCCC2	2.83±0.02	2.83±0.03	2.79±0.02^#^

### Comparison of the test results of method 2 and method 1

There were no statistical differences in IQC detection for the 16 items between methods 1 and 2; hence, method 2 can be used for IQC detection.

### Comparison of the test results of method 3 and method 1

There were statistical differences in the IQC results between methods 1 and 3 (Table 2). Hence, method 3 can not be used for IQC detection.

### Turnaround time monitoring with method 2 for autoanalyser quality control

The TAT of the first batch of samples per day (8:00) for the two months (45 working days) was shorter by an average of 35.23 min in IQC using method 2 compared with method 1. The TAT monitoring results are detailed in Table 3. The results indicated that the Kehua Polaris c2000 biochemical analyser has remote operation capabilities and a special QC warehouse, which can realise autoanalysed QC.

**Table 3:** TAT time comparison.

	TAT of IQC with<br>method 1	TAT of IQC with<br>method 2
Number of Tests	50	50
Timequantum	45 d	45 d
Mean	96.45 min	61.22 min
Median	87 (59, 178) min	53 (31, 156) min

### The correction rate of automatic retesting after IQC runaway with method 2

In the two months (45 working days) of IQC detection using method 2, there were 147 out-of-control occurrences in 16 testing items; of these occurrences, 93 were corrected by automatic retesting, with a correction rate of 63.27%.

## Discussion

A fast rate, easy operation, high accuracy and high sensitivity characterise the automatic biochemical analyser. At present, the detection process has essentially been automated. However, IQC still mainly relies on manual operation; therefore, the operators’ human influence will significantly impact the IQC results [5] [6]. The IQC control of the automatic biochemical analyser effectively controlled the problems generated in an actual test process and ensured the accuracy of the test results [7] [8] [9] [10]. Quality control itself is technical, professional work, and quality inspectors are required to have a high level of professional quality and practical operation skills to ensure the smooth and efficient development of the testing work. However, biochemical testing accidents often occur in the actual operation process due to quality inspectors’ substandard professional quality. Abnormalities in biochemical test IQC processes will affect the quality of the laboratory work and reduce the accuracy of the test, resulting in data differences [11] and affecting the clinical diagnostic accuracy [12]. This will significantly impact the smooth development of the testing work, testing efficiency and results [13].

As a standardised supervision mechanism, IQC can monitor and evaluate the test system’s precision and stability and indirectly evaluate the test results’ accuracy, which is an important mechanism for ensuring the quality of clinical test work [14]. The latest IQC rules must focus on the frequency of QC or the number of samples to ensure the detection quality and support the continuous operation of the high-yield analysis system to obtain the corresponding report results; that is, develop the QC product detection frequency according to the number of tests for each item [15]. Such work cannot be completed manually and only relies on the instrument’s IQC function. At present, the single-machine biochemical analyser does not have an autoanalyser QC mechanism, which the automatic assembly line must realise.

The Kehua Polaris c2000 automatic biochemical analyser does include an autoanalyser. The results of this study showed that the TAT of the first batch of samples per day (8:00) for the two months (45 working days) was shorter by an average of 35.23 min in IQC using method 2 compared with method 1, which indicated that the Kehua Polaris c2000 biochemical analyser has a remote operation ability and a special QC warehouse, which can, to some degree, realise autoanalysed QC. The QC products are placed in a manner relevant to the tests required; the following day, the instrument can complete the instrument preparation and QC detection function before staff arrive at the laboratory to carry out testing work. This simplifies the operational steps and greatly reduces the sample detection cycle time. Balık AR et al. [16], using Beckman Coulter AU680 and Roche Cobas 8000 autoanalyser, reported that appropriate specification limits should be defined to examine test methods that meet the objectives for fitness for clinical purposes.

In case of loss of control, the analyser can also automatically retest for QC, and most random errors can be corrected. The results of this study show that the stability of advanced placement of conventional Roche chemicals in the QC warehouse can support the automatic implementation of IQC on the next day.

However, some limitations remain in this study. First, only one automatic biochemical analyser was used in this study, and subsequent analysis using multiple instruments was required to verify the reliability of the experimental conclusions. Second, the correction rate in this study was 63.27%, and some degree of manual intervention was still required to ensure higher accuracy. Finally, the normativity of the operator needs to be ensured to reduce the result offset brought by the operator.

## Conclusion

The Kehua Polaris c2000 biochemical analyser has remote operation ability and a special QC warehouse. Using its autoanalyser QC module to perform IQC did not impact the IQC detection results presented in this study and could shorten the TAT and automatically correct a degree of IQC runaway.

## Dodatak

### Declarations

### Ethics approval and consent to participate

This study was conducted following the Helsinki Declaration. This study was conducted with approval from the Ethics Committee of Shanghai Shuguang Hospital Affiliated with Shanghai University of TCM. Written informed consent was obtained from all participants.

### Consent for publication

Not applicable.

### Availability of data and materials

All data generated or analysed during this study are included in this article.

### Funding

Not applicable.

### Conflict of interest statement

All the authors declare that they have no conflict of interest in this work.

### Author Contributions

(I) Conception and design: Wang YY

(II) Administrative support: Zhang J

(III) Provision of study materials or patients: Shi L

(IV) Collection and assembly of data: Chen C

(V) Data analysis and interpretation: Wang YY

(VI) Manuscript writing: All authors

(VII) Final approval of manuscript: All authors

### Acknowledgements

Not applicable.

## References

[1] Liu S M, Zhou Q, Ma X Y (2011). Compilation of papers of the second China Clinical Microbiology Conference and Microbiology and immunology Sanjiang Forum.

[2] Cong Y L, Qin X L (2004). An introduction to the clinical laboratory.

[3] Lapić I, Rogić D, Ivić M, Tomičević M, Kardum P M M, Erek L, et al (2021). Laboratory professionals' attitudes towards ISO 15189:2012 accreditation: An anonymous survey of three Croatian accredited medical laboratories. Biochem Med (Zagreb).

[4] Han Z, Lin R W, Luo M K (2014). Research on the effect of quality control management applied to blood samples tested. Modern Diagnosis and Treatment.

[5] Chen J F (2014). Methods and measures of quality control management during the whole process of blood sample test. Contemporary Medicine.

[6] Tang N A (2019). Performance verification of the Roche cobas c702 automatic biochemical analyser. Laboratory Medicine and Clinic.

[7] Ji X C (2017). Methods and measures of quality control management during the whole process of blood sample test. Guide of China Medicine.

[8] He W (2017). Analysis of the whole-process quality control measures of biochemical inspection. Gansu Science and Technology.

[9] Zhao Z J, Guo Y F (2008). Clinical application evaluation of Mindray BS-400 automatic biochemical analyzer. China Medical Herald.

[10] Chen M, Qin S, Yang S, Chen H, Lu L, Qin X (2022). Performance evaluation between two automated biochemical analyzer systems: Roche Cobas 8000 and Mindray BS2000M. J Med Biochem.

[11] Zhao L, Liu X L, Sui J (2015). Analysis of quality control measures for clinical biochemical test. Chinese Health Industry.

[12] Ju D Y, Yang C, Zhang L L (2019). Biochemical inspection of internal quality control and method analysis. Chinese Health Industry.

[13] Xu S, Huang X D, Li X H (2016). Study on the function of the quality control mode of clinical biochemistry examination in the laboratory information management system. Clinical Journal of Chinese Medicine.

[14] Xu H Q, Liu Y P, Fu W L, Huang J F, Huang Q (2017). Development and application of auto biochemistry analyser made in China. Chinese Medical Equipment Journal.

[15] Westgard J O, Bayat H, Westgard S A (2018). Planning risk-based SQC schedules for bracketed operation of continuous production analyzers. Clin Chem.

[16] Balık A R, Gücel F (2021). Evaluation of 20 clinical chemistry and 12 immunoassay analytes in terms of total analytical error and measurement uncertainty. Scand J Clin Lab Invest.

